# Keeping generative artificial intelligence reliable in omics biology

**DOI:** 10.1016/j.patter.2025.101417

**Published:** 2026-01-09

**Authors:** Thomas Burger

**Affiliations:** 1 University Grenoble Alpes, CNRS, CEA, INSERM, UA13 BGE, UAR2048 ProFI, EDyP, 38000 Grenoble, France

## Abstract

Generative artificial intelligence can be used to create realistic new data, even for complex real-world processes that cannot be exhaustively modeled: the model is simply learned from preexisting data. Generative artificial intelligence is therefore expected to be a game changer in omics research, where data collection is hampered by considerable experimental constraints. However, it can also “hallucinate”—i.e., create data that are too original to be realistic—which is a critical issue in molecular biology, as hallucinated inferences could have devastating consequences. The author thus explores various use cases to mitigate hallucination-induced risks and to safely unleash the full potential of generative methods.

## Main text

### Generative artificial intelligence context

More than a decade after the breakthrough of artificial intelligence (AI)—which enabled the spread of statistical learning in almost all scientific domains and the emergence of unprecedented applications in everyday life—a similar revolution is ongoing with the advent of generative methods. The first AI revolution made it possible to extract the statistical properties of data collections of overwhelming size (during training) and to then use them to make increasingly accurate predictions or decisions in ever-more-complex settings (during inference). Today, generative methods propose to go beyond data analytics to achieve data synthesis. These methods can seemingly perform creative tasks—e.g., tools are available to write texts in various languages, to synthesize realistic voices or mimic known ones, and to generate images and videos. The impressive results and extensive use of these capacities has raised possibilities in many fields. Today, researchers in nearly all scientific domains are considering how the underlying technology can be leveraged to advance academic research. Of course, omics biology is no exception, with multiple benefits already envisioned. Generated experimental data could spare animals’ lives, reduce experimental costs, save time, and reduce environmental footprint. New research hypotheses could also be generated. And these are just two examples.

### Why hallucinations should be considered carefully

**Figure 1:**
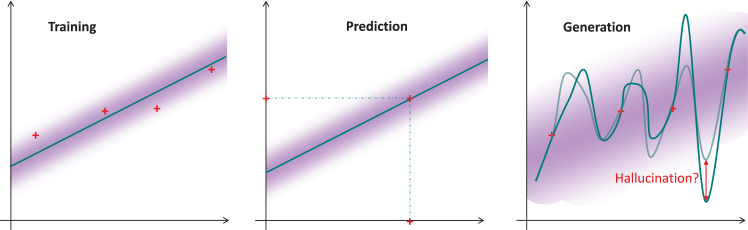
Abstract illustration showing why hallucinations differ from classical prediction errors in machine learning Red points depict constraints (training data, prompts, etc.), teal functions depict the computation results, and the purple shading around these depict uncertainty zones. The training of a model on the left panel conceptually boils down to a regression problem (i.e., an overconstrained problem) where the uncertainty relates to the residual difference between the trained model and the data (i.e., the variance). Performing prediction with model inference, as in the central panel, corresponds to a well-posed problem with a unique solution: the uncertainty does not need to be accounted for, as in its simplest form, prediction amounts to finding the answer that minimizes the residuals—i.e., the mean (or expected) value. During generation (right panel), one solves an underconstrained and ill-posed problem; creative answers make it necessary to step out of the mean trend and to sample answers where outliers can also be found.

Molecular biology researchers have gotten used to handling predictions derived from AI models over the last 10 years,^1^ but they are not yet proficient when it comes to handling generative AI (genAI) outputs. Whereas handling AI-derived predictions essentially amounts to understanding how the training step was conducted to avoid detrimental predictions (biases in training data, possible overfitting, and a couple of other concerns rooted in the bias-variance trade-off),^2^ the risks linked to genAI are more difficult to grasp. As an example, we will take a large language model (LLM) like ChatGPT. An incorrect answer to a prompt (i.e., a hallucination) resembles a succession of incorrect predictions about what the next word to generate should be. However, if we adopt a simplified abstract mathematical viewpoint, as illustrated in Figure 1, things look different.

In machine learning, training a model means solving a mathematical problem akin to the one on the left panel of Figure 1, where a collection of observations, the data points, are used to fit a model. This model is intended to capture the overall trends within the data and to ignore the specificities (as otherwise, it would overfit). The process essentially elaborates on the statistical concept of regression and amounts to solving a problem where the number of constraints is larger than the number of degrees of freedom. From the model, inferences can be made, as depicted in the center panel, simply by estimating the model output based on the input. During inference, the number of constraints equals the number of degrees of freedom. As a consequence, the solution is usually unique and simple to compute. Its correctness, however, depends on the training. The generation problem, by contrast, amounts to the case where the number of constraints is smaller than the number of degrees of freedom (right panel), leaving room for improvisation, for creativity. Because an LLM can exploit these unconstrained degrees of freedom, it can provide different answers to the same prompt. However, doing so is not as easy as it looks. In mathematics, this translates into an ill-posed problem, which is more difficult to solve than performing regression or prediction. Concretely, on the right panel, two distinct curves respect the constraints imposed by the red data points, but between those points, the curves have bumps of varying sizes. If the bumps are reduced to the limit where the curve only interpolates the points, we will come back to a well-posed problem, which will have a unique solution—with two similar prompts yielding similar answers—and the LLM will no longer be creative. Conversely, if the bumps oscillate too much, creativity can become hallucination: something that looks correct (because it fits the constraints) but contains unrealistic details. In other words, the capacity to hallucinate is integral to the creativity that makes genAI interesting; it is simply a matter of magnitude.

Capturing the beneficial or detrimental consequences of hallucinations should not be so difficult when the tool is an LLM. As language is intrinsic to human cognition, any of us should be well equipped to keep a critical eye on hallucinated/creative texts. However, LLMs’ capability to fabricate facts or references has motivated the investigation of retrieval-augmented generation^3^—i.e., sourced answers. Likewise, LLMs are classically endowed with a “temperature” parameter, making it possible to adjust the model’s creativity. However, this parameter appears to be difficult to adjust, and refined analysis indicates that its relation to creativity is weaker than expected.^4^

Another domain where our critical eye is probably as sharp as when assessing text is with natural images. A hallucinated image displaying a hand with an incorrect number of fingers is unlikely to fool a vigilant person. Nevertheless, if the hallucination relates to a more technical image (e.g., histopathology or immunostaining), it becomes more difficult to detect. While the underlying AI models differ between languages and images, Figure 1 remains insightful. Recognizing an object in an image requires having learned its main features and predicting the likeliest tag accordingly. However, generating an image requires going beyond the mean trend (conditioned by the main features or by the prompted instructions) and performing super-resolution—i.e., enhancing the image through realistic addition of the missing details.

These examples show that dealing with the creativity-hallucination continuum is more difficult than the classical bias-variance trade-off that has long bounded the risks linked to machine learning-based predictions. Possibly, future genAI architectures will solve this, but I tend to believe it is inherent to the creativity expectations. Predictive models can focus on the mean trend of the data because it is the quantity that minimizes the residuals (model uncertainty, generalization error, etc.), but genAI must explore the entire distribution. Unfortunately, as the probability of outliers smoothly increases when moving away from the mean trend, the boundary between acceptable (creative) and unacceptable (hallucinated) answers is unclear.

Having this in mind is necessary when discussing the benefits and risks of genAI in a domain like molecular biology. We should not assume that any expert can easily discriminate between a slightly hallucinated omics profile and an odd but realistic AI-generated one. It is thus imperative to examine how these difficulties could translate when data produced by genAI methods are used to explore molecular biology and health—i.e., domains where our understanding is challenged daily. Considering the enormous expectations, the point is obviously not to advocate against the use of these systems, with their potential positive impacts, but rather to find ways to mitigate the risks of spoiling the results due to undetected hallucinations. As part of this approach, I will now present a series of use cases where risk-mitigation policies could be deployed and leverage them to extrapolate some conceptual guidelines.

### Generating hypotheses: Use cases

The first series of use cases pertains to situations where data generation *per se* is not the goal but only a proxy for a more human-driven task: new hypothesis generation.

Prescreening is the most straightforward application of genAI-driven hypothesis generation. The typical examples are protein design and drug discovery with tools like AlphaFold,^5^ which are already used daily. Rather than testing a huge number of putative protein sequences, with prescreening, we can discard those that are likely to have a 3D structure incompatible with the docking constraints. The remaining candidates will then be tested *in vitro* and *in vivo*, following the validation protocol developed before AlphaFold changed the game. The expected efficiency gain is very high. The expected loss relates only to candidates that are erroneously discarded during prescreening, and this risk has long existed in various forms. Beyond the example of its use with AlphaFold, prescreening is an important genAI use case that will likely spread.

Digital twins (DTs) provide a conceptually related use case. A DT essentially refers to a high-fidelity numerical simulation of a real-world process, but with important time-related constraints: it works in real time and accounts for the entire life cycle (notably, it mimics aging). These constraints mean that DTs are not entirely suitable for use with complex biological organisms; by contrast, they are envisaged for simpler ones (e.g., “virtual cell”), and they are ideal for complex biotechnological setups like bioreactors. In that scenario, a DT fulfills many purposes: monitoring, predictive maintenance, training sessions, process optimization, etc. Among them, process optimization can probably leverage the most from genAI. In this use case, genAI can simulate the influence of small adjustments to various parameters, either to improve on current settings (i.e., by tuning parameters to get closer to an implicit optimal point) or to test reliability (i.e., do small perturbations of some parameters lead to different steady states or to unstable behavior?). Like in the prescreening use case discussed above, the idea is to explore a large number of possible configurations at low cost with the aim of finding a “better” one. Admittedly, hallucinations could lead to deterioration, but the risk should be mitigated by an expert with a critical eye who uses genAI only to prescreen the parameter-tuning landscape.

Counterfactual analysis is the third type of hypothesis-generation use case. The main goal of counterfactual analysis is to propose putative answers to “what if” questions that cannot be tested in real life. For instance, what if patient no. 12345 had not died in a car accident during the trial? Would the tested drug have led to a cure for her? Counterfactual analysis is an old field of statistics that was specifically developed to answer this type of question. This field has witnessed a recent revival with the rise of explainability challenges linked to deep learning predictions.^6^ In this context, a counterfactual explanation expands on the smallest change in feature values that can lead to a distinct outcome. Whether one needs to explain a deep learning decision or why a drug may or may not work for a given patient, the line of reasoning is the same and genAI will prove useful. Like the DT use case discussed above, counterfactual analysis makes it possible to generate a huge amount of similar data, with small perturbations, at low cost. These data can then be mined for putative counterfactual explanations, among which only the most likely ones will be retained for full investigation.

A feature common to the DT and the counterfactual analysis use cases is the need to efficiently test many minor perturbations of a given system as to find the small number that trigger different outcomes and that are consequently worth investigating more thoroughly. This idea has recently emerged through the concept of large perturbation models,^7^ and efforts in developing them in the future will directly impact how academic research can turn genAI-aided.

In the three hypothesis-generating use cases presented (prescreening, DTs, and counterfactual analysis), the degree of hallucination essentially translates into a degree of relevance. Thus, if the data generation is particularly accurate, the scientist can extract a highly relevant new hypothesis, potentially triggering groundbreaking improvements (the DT example) or research avenues (prescreening and counterfactual analysis). However, in case of hallucination, the new hypothesis will be fanciful, which is a pity but is not really more damaging than following erroneous human intuition (e.g., from a not-so-great student) or untrustworthy data (e.g., from an article that is later retracted). In the worst case, the principal investigator may lose some time and waste some funds by testing the irrelevant hypotheses, but this should not translate directly into hallucinated biological conclusions.

### Generating experimental data for biological investigations: Use cases

We now turn to a second series of use cases requiring the generation of data itself. The aim is to replace real experimental data with *in silico*-generated data. As sketched out in the “generative artificial intelligence context” section, the benefits of this approach are manifold. However, in this context, hallucinations can have important downsides. Therefore, I present three use cases where these damaging consequences can be controlled.

First, we will consider null hypothesis data generation. In statistics, “null data” refers to measurements that are common and should not lead to a notable fact (aka a discovery). These data are often conflated with cases where the *p* value is not small enough, meaning that they are not retained for subsequent investigations.^8^^,^^9^ Classically, to compute a *p* value, one relies on the data to be tested and on a model of the null data (e.g., Student’s *t* distribution for a *t* test). However, for complex biological data, we do not always have a ready-made null mathematical model. An alternative is to rely on an empirical null model. To illustrate this, recall when the first next-generation sequencers appeared. At the time, it was not trivial to statistically assess the significance of the differential expression of gene-associated transcript read counts because the negative binomial model was not that popular. Instead of using an ill-adapted statistical test (e.g., a *t* test), it was more appropriate to perform empirical null testing. To do this, the null model could be derived from random permutations between the samples^9^ or through computation of gene-wise summary statistics for two cohorts of healthy patients. Another example is that of peptide identification in mass spectrometry-based proteomics. To assess the significance of a spectrum match to an amino acid sequence, researchers classically rely on “decoy matches.” Artificial random spectrum-sequence pairs are generated from amino acid sequences that should not be found in the samples analyzed. Taken together, the summary statistics of these random pairs yield a null model.^9^ A last common example is from single-cell transcriptomics. If a classical statistical test is performed on the same genes as those used to cluster the cells into several cell types, it will lead to an artifact referred to as the double-dipping problem.^8^ In this scenario, too, null hypothesis generation is an alternative. In all of these cases, null data generation is sought and genAI offers incredible opportunities as long as hallucinations do not deteriorate the subsequent statistical inferences. Fortunately, a natural risk-mitigation policy is available because data are generated only under the null hypothesis: if one assumes the data are not obviously biased (which in itself is a concern, but this is not specific to genAI), more hallucinations should essentially lead to more diversity in the null data, which will reduce the statistical power rather than lead to false discoveries.

A second possible use case is negative control data generation. Such data are also linked to the concept of the null hypothesis; however, instead of data being directly generated under the null hypothesis, observations are generated to define the null hypothesis. The difference can be illustrated using differential analysis of gene expression. In the above use case, one generates the summary statistics resulting from testing nondifferential genes. By contrast, here, the aim is to generate realistic read count profiles, which should not be differentially abundant—e.g., when measuring control samples (healthy patients, wild-type samples, or whatever). The reason for generating this type of data stems from the old idea of sharing or reusing control samples in experiments where the tested condition is different. Although test conditions change constantly, the control samples should remain similar. It therefore makes sense to leverage the huge amount of control data that have already been acquired to train different models (tailored to the experimental designs and conditions) to accurately generate new control data. This approach has not broken through in omics science so far. Nevertheless, tools are progressively emerging, such as Evo 2,^10^ which can generate realistic genomics data. In the near future, those tools may become accurate enough to generate sufficiently realistic control samples with which to compare test samples.

Finally, it is possible to leverage genAI for variability-preserving data modification and for adaptation of classical machine learning models to fit new contexts. Data modification is a bit more technical to implement, but various scenarios are already well described, among which are privacy preservation, domain adaptation, and missing value imputation. For privacy preservation, the objective is to modify the data sufficiently to make deanonymization impossible. For instance, assume that patients’ names are replaced by noninterpretable IDs, but their unique combination of clinical and genetic features makes them nevertheless identifiable, so that a malicious hacker could exploit the information for personal gain. A workaround is to sufficiently modify the data to make sure the patients’ profiles cannot lead to identification while guaranteeing that the modifications made are small enough for the overall data distribution to remain unchanged and therefore still compatible with statistical analysis.^11^ Domain adaptation, by contrast, aims to translate the data to fit a new context, where data are not as easy to access.^12^ Finally, missing value imputation corresponds to the classical case where a few measurements are missing: instead of the incomplete samples being discarded, the datasets can be modified to look complete. The challenge is to do so as accurately as possible without affecting downstream analysis. Data imputation has long been accounted for in classical regression settings (researchers try to find the most likely prediction for the missing value), but genAI has already been identified as a valuable addition in the field of single-cell transcriptomics.^13^

In these three data-generating scenarios (null hypothesis generation, negative control data generation, and variability-preserving data modification), the artificial data are used directly to perform statistical analyses and draw biological conclusions. However, it is possible to stick to risk mitigation with respect to possible hallucinations by using genAI’s creativity to increase the diversity of the reference data (control samples or observations falling under the null hypothesis). When this is done, exaggerated hallucinations should lead borderline samples to look like mainstream samples rather than lead to false positives.

### Reusing data for methodological development in bioinformatics: Use cases

For the last series of use cases, we will look toward a future where genAI tools are customary in various areas, including omics science. This widespread availability will undoubtedly lead us to reconsider our relationship to experimental data. Although experimental validation will remain the gold standard for some time yet, we can already safely assume various scenarios where it will not be as necessary, where generated data can be conveniently used as a substitute. This is particularly relevant for the development and fine-tuning of new bioinformatics methodologies.

The first scenario is obviously that of extensive simulation. With cheaper, more accessible, and more realistic data generation, various tasks are facilitated: benchmarking, validation with ground truth data, stress tests, sensitivity analyses, etc. Moreover, and following a line akin to the “data modification” use case described above, easy access to generated data will make it possible to improve the robustness and scope of machine learning-based prediction models. Beyond the aforementioned domain adaptation case (where shifts to slightly different contexts can be accounted for despite less associated data), genAI has already been highlighted as useful for bootstrapping and data augmentation.

Bootstrapping refers to a statistical technique where the original data points are used to estimate a distribution, which is in turn used to generate new data items. These new items can then be incorporated to refine the statistical process. Although it might look like magic, like pulling oneself up by one’s own bootstraps, the statistical correctness of the procedure largely depends on how the new data are generated.^14^

Data augmentation encompasses a wide set of techniques to artificially inflate the size of the datasets used in deep learning models (which tend to require many training examples). One classical technique is to add artificial data to the real dataset or to create various modified forms of each data item by relying on systematic transforms. It is even possible to improve the generalization capabilities of the model by training it on generated data where biases inherent to the data collection process are reduced.^15^

Ultimately, and as proposed by Efros in his MIAI distinguished lecture, genAI will become sufficiently widespread and reliable to serve as a surrogate for data storage. Thus, instead of saving huge volumes of experimental data, with the corresponding considerable memory and energy footprints, we will simply store a model trained on them so as to re-generate them on demand (with some stochastic variations). This approach can be used not just for methodological development but also for all tasks that do not necessarily require new, real experimental data.

In these data reuse cases (extensive simulation, bootstrapping, data augmentation, and data storage), genAI is involved in the development of bioinformatics methodology to improve the resulting software, exactly like any new theoretical breakthrough in mathematics or computer science. As such, the risk of hallucination should not need to be directly accounted for by the molecular biology investigators, but these investigators should simply keep using the same critical eye as when they incorporate a new machine learning-based tool into their investigative workflow.

### Conclusions

Like any technological breakthrough, use of genAI will progressively spread as researchers become acquainted with its control. Becoming acquainted includes acquiring sufficient theoretical knowledge and having accessible software tools but also adhering to principles for correct usage. Here, I have tackled the last point, based on the worst-case evaluation policy, which can be summarized by the following question: how should one use a given genAI tool so that if it produces an undetected hallucination, it will not lead to a false biological discovery (i.e., the scientific counterpart of fake news)? Ten scenarios are listed: prescreening, DT optimization, counterfactual analysis, null hypothesis data generation, negative control data generation, variability-preserving data modification, extensive simulation, bootstrapping, data augmentation, and data storage. However, this list is not exhaustive. It would benefit from the addition of “safe” use cases that will undoubtedly appear in the scientific literature in coming years. Hopefully, this will lead researchers to adopt a critical eye when viewing genAI data, as with experimental data, so it becomes part of the scientific approach.

## Acknowledgments

The author is grateful to Magnus Palmblad, Robbin Bouwmeester, and Lukas Käll for organizing the 2025 Lorentz workshop on AI Methods for Planning and Interpretation of Proteomics Experiments; to Frédéric Bertrand, Oscar Vassalo, and Marina Pominova for fruitful discussions during the workshop; and to the anonymous reviewers for their valuable comments. The author’s research was supported by grants from the French National Research Agency: ProFI project (ANR-10INBS-0008), GRAL CBH project (ANR-17-EURE-0003), France 2030 program (ANR-19-P3IA-0003), PeptidOMS project (ANR-24-CE45-3296), and ProteoVir project (ANR-24-RRII-0001).

## Declaration of interests

The author declares no competing interests.
